# Impacts of Social Inequality, Air Pollution, Rural–Urban Divides, and Insufficient Green Space on Residents’ Health in China: Insight from Chinese General Social Survey Data Analysis

**DOI:** 10.3390/ijerph192114225

**Published:** 2022-10-31

**Authors:** Peng Zhou, Siwei Sun, Tao Chen, Yue Pan, Wanqing Xu, Hailu Zhang

**Affiliations:** 1 School of Civil Engineering and Architecture, Wuhan Institute of Technology, Wuhan 430074, China; 2 School of Arts and Communication, China University of Geosciences, Wuhan 430074, China; 3 Hubei Planning, Design and Research Institute, Wuhan 430064, China

**Keywords:** health, social inequality, air pollution, travel behavior, urban–rural differentiation mechanism

## Abstract

Attention to physical and mental health is becoming more intensive. In China, factors and mechanisms are now a focus of research. We used dynamic air quality monitoring data and the Chinese General Social Survey (CGSS) to assess the spatial differences and the coupling between subjective and objective air pollution. In addition, a logistic model was used to explore the impact mechanisms of social inequality, air pollution, food safety, and lack of green space on health. The results show that (1) the impact of subjective and objective air pollution on the health level of the population is significant; (2) income inequality, air pollution, food pollution, and travel behavior significantly affect the residents’ health; and (3) environmental health has a significant differentiation mechanism between urban and rural areas. The negative health effects of air pollution and insufficient green space are more significant in cities; food pollution is more likely in rural areas. In terms of socioeconomic inequality, gender, family size, travel, and physical exercise had no significant effect on rural health. Health improvement was higher in the low-income group than in the high-income group. The adverse effect of travel behavior on environmental pollution is conducive to improving health. Therefore, social equality, strictly controlled environmental pollution, exercise, and travel can help narrow the gap between rich and poor, promote urban–rural health equity, and improve human health.

## 1. Introduction

In the current era of comprehensive health, China’s demand for physical and mental health has stimulated the national demand for health research, health improvement, and research on influencing factors and their mechanisms of action based on Chinese samples. Many international scholars have used walkability as a mediating variable to discover the health effects of social injustice and green space and the effects of environmental pollution on physical and mental health, gradually forming a complete system that covers sociology, economics, geography, behavior, public health, and psychology. However, a complete interdisciplinary system has yet to be developed in China to explore the factors influencing human health. Therefore, the relationship between social inequality, environmental pollution, insufficient green space, and health still needs to be explored.

Although social and wealth inequality have often been key elements in studying human health influences, environmental pollution, lack of blue-green space, and preferences for travel behavior have been neglected. This situation occurs because dynamic monitoring of environmental quality, blue-green space scale, quality assessment, behavioral tracking surveys, monitoring in micro-social surveys, and macro panel data analysis have remained limited by spatial technology and monitoring techniques. However, the importance of the impact of environmental pollution on health has been empirically demonstrated in the fields of sociology, environmental studies, urban planning, and geography [1,2,3]. Studies have been conducted to overcome the limitations of data acquisition and scale accuracy to include air pollution in the independent variable indicator system of regression models to explore the negative environmental effects of air pollution on physical and mental health [4,5,6]. Since 2010, the effect of green spaces on health has been a popular research topic, and the number of such studies has grown. Many international scholars have identified the health improvement effects of green spaces using walkability and the frequency of physical activity as mediating variables [7,8,9]. However, in China, research on the mechanism of the effects of blue and green elements and behavioral preferences on health remains in the early stages of development. Gradual empirical evidence of the impact of behavioral characteristics on health has also become a current research hotspot in behavioral and health geography. Scholars in related fields have actively explored the impact of dietary behavior on obesity signs [10], the health impact of travel behavior and exposure to pollution [11], the impact of environmental behavior on health economic effects and pollution sensitivity [12], and the avoidance effect of travel behavior on environmental pollution [13].

Combining the relevant literature on the exploration of health-influencing factors shows that mathematical statistics and spatial models including correlation analysis [14], multiple linear regression models [15], logit [16] and logistic [17] models, probit models [18], and other artificial intelligence models [19] are primarily used to dissect the coupling relationship between social inequality, air pollution, inadequate green space, and health, and their mechanisms of action. Simple multivariate linear models can no longer meet the needs of discontinuous categorical variables as explanatory or explained variables, which has led to a series of logit and logistic models and empirical evidence of their robustness and feasibility of multivariate marginal utility exploration. Shi and Yanwei [20] constructed ordered logit models to explore the mechanism of leisure behavior characteristics on women’s leisure satisfaction. Recently, another improved model with a multistage and multi-breakpoint regression design was applied to explore the associated mechanisms and feedback effects among environmental pollution, social inequality, and health and well-being. Multistage associative least squares (3SLS) have been applied to the coupling between environmental quality and wealth inequality from a health perspective. Liu Cong et al. [21] empirically tested the effect of air quality deterioration due to air pollution on the urban–rural income gap using a system of associative equations. The two-stage probit model is frequently applied to explore intrinsic links between different variables. Ma Xiaojun et al. [22] used this model to discover the factors that influence happiness and the feedback effect of happiness on environmental behavior. The categorical variables of the social surveys further require applied spatial regression models to explain the mechanisms of their explanatory variables as fully as logistic models in the factors exploration of the influencing health and their mechanisms of action.

Many previous studies have focused on objective environmental pollution but have neglected subjective environmental pollution. In recent years, some studies have gradually revealed the endogeneity of subjective and objective air pollution and well-being, finding that subjective air pollution has a more significant influence on well-being, life satisfaction, and psychological health [23]. When exploring the effects of environmental pollution variables such as air pollution on human health, a clear distinction should be made between subjective and objective levels of environmental pollution. Different studies have used various subjective and objective indicators to measure health levels, but significant heterogeneity has also been observed between self-rated and objective health. Self-rated health is a subjective assessment that focuses on physical and mental health. Meanwhile, objective health can be reflected in many public health statistics such as mortality, morbidity, mortality from air pollution-related diseases, and neonatal mortality. This study identified the variability between subjective and objective air pollution. Furthermore, this study explored the mechanisms of social inequality, air pollution, and insufficient green space, among other factors, on health using logistic models. This study aims to promote the exploration of environmental pollution heterogeneity and its impact on health and life from subjective and objective perspectives in the fields of environmental studies and sociology.

## 2. Material and Methods

### 2.1. Data Sources

This study was based on the Chinese General Social Survey (CGSS) Database. Given that the CGSS environmental survey on the ‘severity of the following types of environmental problems in your area’ has not been available since 2013, this study used data from the 2013 CGSS to investigate the impact of the mechanisms of environmental problems and social inequalities on health. The data were cleaned to exclude those who answered ‘do not know’ to the environmental questions related to air pollution, lack of green space, degradation of arable land quality, and food pollution (i.e., excluding samples with missing items in the environmental pollution assessment variables) and removed extreme outliers. The final study was conducted using 5966 valid samples.

### 2.2. Variable Selection

Individual socioeconomic characteristics have historically had a significant influence on health [24]. However, annual income, gender, and age have been primarily used to distinguish disadvantaged groups from the general population, with some studies neglecting the influence of household characteristics and living space. Class and educational levels are often missing from panel data studies due to their limitations. In recent years, wealth and class gaps have received increasing attention. In the past, the simple use of individual income to measure class disparity was inaccurate. This study used individual self-rated class levels from social surveys to assess the impact of social class disparity on health.

Considering that the dependent variable ‘health level’ was selected based on the ‘subjective self-assessment variable’, two variables were available in the social survey: survey question a15, ‘How do you feel your current physical health status’, and question a16, ‘How often in the last four weeks have health problems affected your work or other daily activities’. As the latter is a more accurate indicator of the impact of health problems on daily work and life and a more accurate assessment of health levels, it was chosen as the dependent variable in this study. Furthermore, 0 = always, often, and sometimes, so health problems affect work or life, and are defined as ‘0’ in the unhealthy category, whereas the others are defined as ‘1’ in the healthy category.

In terms of independent variables, socioeconomic variables included annual income, age, marriage, class, education, gender, household size, and housing area per capita; location variables included urban–rural and east–west sub-regions; activity preference variables included the frequency of physical exercise and travel mode; environmental pollution variables included air pollution, water pollution, noise pollution, food pollution, lack of green space and degradation of arable land quality, as shown in Table 1.

### 2.3. Statistical Description

Descriptive statistics of the data are shown in Table 2. In terms of socioeconomic variables, the annual income level of all individuals interviewed was approximately CNY 8365; the mean age was approximately 46 years; and the level of self-assessed social class was low. Furthermore, family size, educational level, and housing area per capita were small. In terms of urban–rural distribution, rural samples were predominant. In terms of activity preferences, physical exercise was infrequent, and the mode of travel was not in line with ‘always take a taxi or private car when going out’. Regarding environmental pollution, the quality of the air environment varied greatly due to the vast size of China.

### 2.4. Model Setting

Objective air pollution was represented by the annual average concentration of PM2.5 (particulate matter 2.5 μm or less) in 2012, using air quality monitoring data. Meanwhile, subjective air pollution was evaluated using the survey question a16, ‘How often did health problems affect your work or other daily activities in the past four weeks’ from the CGSS database. Then, the ratings were assessed. The correlation coefficient was used to explore the correlation between the subjective and objective air pollution variables. Then, subjective air pollution and objective air pollution were introduced separately into the logistic regression model to assess the effect of both on the health levels using the significance of the variables.

## 3. Empirical Analysis

### 3.1. Coupled Relationships between Social Inequality, Air Pollution, Lack of Green Space, and Health

**Figure 1:**
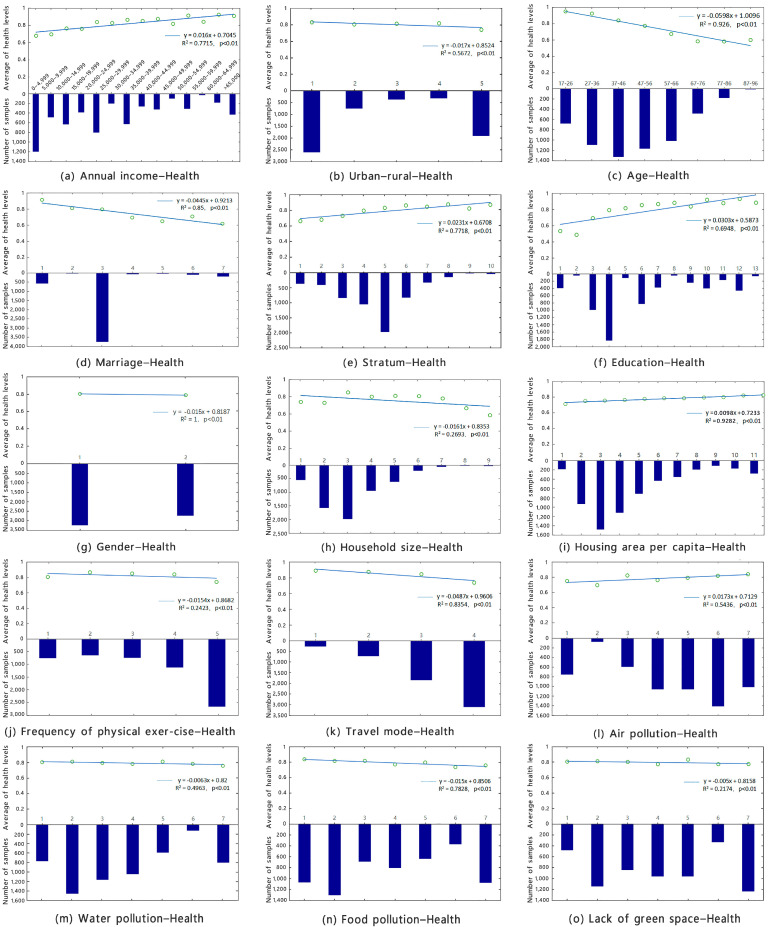
Coupling of variables with the health levels.

First, basic statistical analysis charts were used to explore the coupling relationships between the explanatory variables and health. In the results of the statistical analysis, the health level varied with different individual social characteristics, economic level, travel preferences, and environmental pollution with the corresponding linear trends, as shown in Figure 1.

The response variable in this subsection of the statistical analysis is the mean level of health. The explanatory variables are the values of each statistical survey variable. Frequency distributions, scatter plots, and linear trend line distributions in the statistical analysis adequately express the degree of clustering of the sample in different characteristic groups and the direction of correlation. Furthermore, the slope of the trend line expresses the magnitude of the change in mean health level caused by the explanatory variables.

#### 3.1.1. Age

Sample frequencies with different age distributions were mainly concentrated in the young and middle-aged groups. The degree of health was negatively correlated with age (i.e., the younger the person, the higher the degree of health). Figure 1a demonstrates differences in the magnitude of change in health levels for different age groups, with a smaller decline in health levels between 17 and 36 years and a larger decline in the average health level above 37 years. Specifically, the decline in health levels with age in the young adult group was much smaller than in the elderly group. The histogram in Figure 1a shows that the population age distribution had a clear trend toward a normal distribution, with the largest number of people in the 37–56 age group and the smallest number of people aged 77 years and older.

#### 3.1.2. Annual Income

Annual income was directly proportional to self-rated health, with higher income associated with higher health (average health level). In Figure 1b, the number of people with an annual income between CNY 0 and CNY 4999 was higher, with the next highest number of people in the CNY 20,000–CNY 24,999 range. The slope of the trend line was 0.016 with an increase in annual income, which was less than the slope of the age–health trend line, indicating that the average level of health decreased less than the age–health range with a decrease in annual income.

#### 3.1.3. Number of Family Members

The number of household members was positively related to self-rated health, with higher income being associated with higher health. The number of family members was related to self-rated health in an ‘inverted U-shaped’ curve, as verified by a polynomial. In Figure 1c, the frequency distribution of family members and health levels in this survey sample showed a normal distribution trend, with the highest proportion of ‘three-member families’ and ’two-member families’ up to 50%.

#### 3.1.4. Marriage

Marriage was inversely related to self-rated health, and married people had a higher level of health than unmarried individuals. In Figure 1d, the highest number of people were married to a spouse, followed by the second-highest number of single people. The slope below the marital status health trend line indicates that the decline in health levels with marital separation was higher than the marital health margin.

#### 3.1.5. Education

The level of education was positively related to self-rated health, with higher levels of education associated with higher levels of health. The frequency of the sample distribution gradually increased from ‘no education’ to ‘junior high school’ and then decreased with increasing level of education, which follows the general trend of the distribution of education in China. The increase in the average level of health in these two stages was as follows: before junior high school, the health level increased rapidly with the increase in education level (from 0.5 to 0.77 through three levels of education), whereas after junior high school, the health level slowly increased with the increase in educational level (from 0.77 to 0.97 through nine levels of education) from vocational high school to postgraduate level and above.

#### 3.1.6. Mode of Travel

When travelling, the level of compliance with always taking a taxi or private car decreased from 1 to 4, with 1 being ‘very compliant’ and 4 being ‘very non-compliant’. Figure 1f shows that the mode of travel was inversely related to health, with those who constantly travelled by car having the highest level of health. Thus, exposure to environmental pollution and travel time by car must be reduced.

#### 3.1.7. Exercise Frequency

The frequency of exercise decreased from 1 to 5, with 1 being ‘every day’ and 5 being ‘never’. Figure 1g shows that the frequency of exercise was negatively correlated with the level of health (i.e., the more frequent exercise, the higher the level of health, and vice versa).

#### 3.1.8. Air Quality (Air Pollution)

The air environment quality (air pollution) variable gradually decreased from 1 to 7, as shown by the air quality–health level trend line. The less polluted the air, the higher the level of health.

#### 3.1.9. Housing Area Per Capita

Housing area per capita health statistics showed that the housing area per capita variable was positively correlated with health.

## 4. Results

### 4.1. Modeling Results

Logistic regression models differ from other general linear regression models in terms of simulation accuracy. Linear regression typically uses R-squared values to assess the accuracy of regression results. Meanwhile, logistic regression models use sample prediction accuracy, receiver operating characteristic (ROC) curves, and Akaike information criterion values. The ROC curve is a comprehensive assessment of the degree of correct prediction for different samples and is mainly expressed in terms of the area under the curve (AUC). The larger the AUC, the higher the accuracy of the model regression results and vice versa. Based on the comparison of the model results in this study, the competent full-sample air pollution model had the highest accuracy, whereas the full-sample objective air pollution model had the lowest accuracy.

According to a series of logistic and logit regression model simulation accuracy comparison studies, the perceptibility curve (ROC curve) is based on a comprehensive analysis of a series of indices such as the accuracy rate, true positive rate, false positive rate, and recall rate to assess the equation simulation accuracy, but it still needs to be combined with the Akaike information criterion (AIC) to further verify the model fit goodness. Examination of the AIC value can explore the causal relationships and mechanisms of action between the independent and dependent variables with a minimum number of free parameters to complete. In many regression analyses, increasing the number of free parameters can improve the accuracy of the model but can also easily result in overfitting. AIC is another method to validate the optimal fit of a model proposed by statisticians to address this problem. The AIC can be expressed as −2log(L) + 2p, where k is the number of parameters, L is the log-likelihood, n is the number of observations, and p is the number of variables in the model. It is assumed that the model errors follow an independent normal distribution. The red pool AIC is inversely correlated with the goodness of fit of the model (i.e., the smaller the AIC, the better the model simulation accuracy). A comparison of the model results showed that the simulation accuracy of the full-sample objective air pollution model was better than the full-sample competent air pollution model.

An overview of previous studies revealed that the multiple independent variables in the modeled prediction equations were mostly related to each other and were not entirely independent of each other. Spatial regression measures the strength of this linkage using multicollinearity, which is measured using the variance inflation factor (VIF) or tolerance, with an inverse correlation between VIF and tolerance. A higher VIF or lower tolerance indicates substantial multicollinearity between the variables. According to relevant statistical studies, a VIF <0.5 or tolerance >0.2 proves that there is no multicollinearity between the variables in the equation (i.e., the structure of the variables in the equation is reasonable). The results of the model simulations in this work, which used stepwise regression, showed that the VIF of each variable was <0.5 after several iterations, indicating minimal co-linearity between the explanatory variables in the health impact factor exploration equation; that is, the results were scientifically reasonable.

### 4.2. Interpretation of Model Variables

The results of logistic regression model 1 show that the 10 variables—urban rural, class, household size, education, income, area per capita, lack of green space, food pollution (food safety), east–west, and subjective air pollution—have a significant positive effect on health levels in Table 3. The four variables of gender, age, frequency of physical activity, and mode of travel showed a significant reduction effect on the level of health. The regression parameters of model 1 showed that the probability of women’s health improving relative to that of men decreased by 14.18%, whereas the probability of health deteriorating increased by 4.50% if the age increased by one year. As all of the respondents in this study were a sample of people aged 17 years or older, they avoided the need to consider requirements such as breakpoint regression pre-processing for underage and adult populations. The results showed that the linear negative effect of aging on the probability of improved health is scientifically justified. The mechanism of gender and age further indicates that the negative health effects of disadvantaged groups (women and elderly) are more pronounced. Therefore, in the process of residential planning, environmental improvement, and the layout of facilities, the sensitivity of the living environment and the health improvement needs of disadvantaged groups need to be further considered, which is an inevitable requirement for the development of an aging society. The significant *p*-value for the variable “insufficient green space” further highlights the health-enhancing effect of green space.

The subjective and objective air pollution variables were added to the logistic regression models as input variables, respectively (Table 3—models 1, 2, and 3). The regression results showed that the *p*-value for the subjective air pollution variables was less than 0.01, which had a significant effect; the *p*-value for the objective air pollution variables was greater than 0.1, which meant that they were excluded from the model variables. The evidence shows that the relationship between the objective air pollution levels and subjective air pollution levels can be quantified from the perspective of objective environmental quality monitoring and subjective human perception, and that there is no ‘one-to-one’ relationship between the two, but there is significant heterogeneity because subjective perceptions of air pollution are influenced by differences in individual characteristics. Past empirical studies have shown that at the same level of objective air quality, vulnerable groups such as women and the elderly have higher subjective perceptions of air pollution, that higher income groups are more sensitive to air pollution, and sensitive people are more likely to be affected by air pollution at the perception level.

The regression results from model 4 (urban sample) and model 5 (rural sample) in Table 4 showed that the significance of ‘air pollution and lack of green space’ held for the urban sample, whereas the significance of ‘air pollution and lack of green space’ for the rural sample was greater than. This means that ‘air quality and green space’ have a significant impact on the health of urban residents. In terms of ‘food safety’, the coefficient for the urban sample was smaller than the coefficient for the rural sample, indicating that rural residents are more likely to experience negative health effects when exposed to food contamination, thus suggesting that rural residents are at greater risk of food contamination exposure.

## 5. Discussion

### 5.1. Analysis of the Mechanism of the Impact of Environmental Pollution on the Health Level of the Population

#### 5.1.1. Subjective and Objective Air Pollution

For ambient air pollution, many studies in the past have stayed at the level of objective environmental pollution air quality, thus neglecting subjective environmental pollution [25]. Objective air pollution is a direct expression of air quality monitoring data, often proven to be directly related to human health and is influenced by certain factors such as vehicle emissions and urban morphology [26,27,28]. This series of studies is based on objective environmental pollution and ignores subjective environmental pollution. Several studies have gradually revealed the endogeneity of subjective and objective air pollution and well-being, finding that subjective air pollution affects well-being, life satisfaction, and psychological well-being to a more significant degree.

Individual differences in environmental sensitivity lead to differences in subjective evaluations and objective measures of environmental pollution, so subjective air pollution levels can show significant differences between individuals. Objective air pollution is indicated by the actual pollutant composition and pollution indices monitored. From these actual measurements, it is possible to infer to what extent objective air pollution threatens physical health at a physical level such as an increased probability of respiratory and cardiovascular diseases, but it is impossible to accurately characterize the reduction in subjective mental health levels associated with objective air pollution. Subjective air pollution is an evaluation of the level of pollution from the residents’ perceptions of the objective air environment. It is a comprehensive expression of the objective measured data and subjective perceptions of experience, and is a comprehensive evaluation of the extent to which air pollution affects physical health and mental health, and is a more scientific assessment of the extent of health effects for the surveyed individuals [29]. The more concerned and sensitive people are about air pollution, the greater their subjective perception of the impact of air pollution levels on their daily lives, and the greater their perception of the health risks. Therefore, subjective air pollution indicators are of greater research significance in the study of the assessment of individual health level and its influence mechanism.

Air pollution affects the health of the population at both the physical and mental levels. The impact of air pollution on physical health is mainly reflected in the triggering of respiratory diseases such as respiratory infections, asthma, chronic obstructive pulmonary disease, and lung cancer, and even long-term exposure to heavy air pollution can raise the mortality rates. Haze can have a significant impact on urban economies, the tourism industry, and residential travel. Subjective air pollution poses risks such as mobility restrictions, safety threats, and health threats, and the subjective risk perceptions that residents generate from the haze can create feelings of stress and anxiety. This range of negative emotions will cause a decrease in mental health.

#### 5.1.2. Insufficient Green Space

Green space has a significant health-enhancing effect. Green space has been a key focus of health research in the past, and studies on the health effects of green space have developed rapidly in the country and abroad in recent years, with a large body of literature focusing on the effects of land use type and green space on physical and mental health. Green spaces can improve the frequency, duration, and willingness of residents to be physically active through walkability, and improve air and habitat quality, which improves physical fitness [30,31]. Furthermore, green views and open green spaces are conducive to reducing stress and tension and improving psychological well-being. This study empirically emphasizes the negative health effects of insufficient green spaces.

Although green space has a significant impact on the health levels of residents, a variety of quantitative approaches and green space variables quantify how to measure the accessibility, scale, quality, and type of green space. In the early literature, the only variable chosen to measure the explanatory variables of green space was ‘accessibility of green space’ [32,33,34]. In later literature, the heterogeneity of ‘size and quality’ of green spaces on resident activity preferences, activity types, and health was gradually considered [35]. The variable ‘insufficient green space’ selected in this study was a subjective evaluation of the adequacy of green space by urban and rural residents from a ‘humanistic’ perspective and a comprehensive evaluation of the ‘accessibility, scale, quality, and type of green space of the residence. It is also a comprehensive evaluation of the accessibility, scale, quality, and type of green space, which is more conducive to reflecting the degree of influence and mechanism of green space on the residents’ health, and provides a new direction for future research on the health effects of green spaces.

#### 5.1.3. Food Contamination (Food Safety)

The ‘food environment’ element is neglected in urban planning, geography, and health geography studies. This study fills the gap between these fields and their related intersections: ‘urban residents’ subjective perceptions of food contamination and how it affects health levels. In the current era of health, there is an urgent need to improve the quality of the food environment. This study measures the “quality of the food environment” through the variable “food safety”, which provides a side-effect on the severity of “food desertification”. Food safety factors affect human health through accessibility and food availability and have a clear urban–rural divide.

### 5.2. Analysis of the Mechanisms of Socioeconomic Inequalities and Urban–Rural Differences on the Health Level of the Population

Socioeconomic inequalities lead to significant mechanisms of differentiation in their individual health. Urban rural, income, class, household size, education, and housing space per capita reflect the social class differences, wealth disparities, and urban–rural disparities from the side as well as feedback on the socioeconomic conditions and mechanisms acting on health from a social inequality perspective. The urban–rural divide had a significant impact on health, with cities having a 20.8% higher probability of improving the health of the population than rural areas, an increase that was higher than other influencing factors; highlighting that cities outperform rural areas in terms of health improvement. Increasing incomes, increasing classes, and educational attainment bring better access to health care, housing, and infrastructure, which are beneficial to health improvement, with probabilities of 34.18%, 17.35%, and 6.18%, respectively. This finding shows that improvements in urban/rural income and class levels can cause qualitative changes in health.

Differences in air quality and green space between urban and rural areas led to prominent differences in the health levels of urban and rural residents. The overall health level of urban residents was higher. The overall health level of urban residents was higher than residents in urban–rural areas and rural areas. There were significant differences in the characteristics of urban and rural habitats, with urban areas densely populated and industrially active whereas rural areas were sparsely populated and mainly agricultural, resulting in lower concentrations of air pollutants and lower air mobility in urban areas than in rural areas, increasing the concentration and duration of exposure to urban air pollution for people, which led to the health effects of air quality being more pronounced in cities. The high density, spatial compactness, and fast pace of urban life increase the demand for air quality and green space by urban residents compared to the slow-paced and low-density rural areas, further increasing the extent to which both environmental factors affect the health of urban populations. Thus, there is a clear mechanism of urban–rural locational differentiation in the unequal health effects of air pollution and insufficient green space, with both having a more significant negative health effect on urban populations. The health level of urban residents was more significantly influenced by two types of environmental factors, namely air pollution and insufficient green space. Thus, urban residents are more concerned about their health from the perspective of environmental quality, and urban residents are more at risk of being affected by air pollution and insufficient green space factors. Furthermore, the evidence shows that urban residents spend more on health in response to environmental pollution than rural residents.

For each unit increase in income, the health improvement of rural residents is much greater than that of urban residents, which shows that the impact of the urban–rural income disparity on health is significant. In recent years, the gap between the rich and the poor in China’s urban and rural areas has gradually narrowed but still exists, and the overall income level of rural residents is lower than urban residents; however, this study showed that the health improvement of the low-income level group with increased income was much higher than the high-income level group, further proving the practical significance of rural revitalization and precise poverty alleviation in China. In contrast, the effects of gender, household size, travel, and physical activity on the health of the rural population were not significant under the perspective of socioeconomic inequality.

### 5.3. Analysis of the Mechanism of Activity Preference and Travel Behavior on the Health Level of the Population Activity Preferences and Travel Behavior Directly Affect Health

Daily physical activity significantly improves health, whereas never travelling mechanically increases the time and level of exposure to environmental pollution and reduces self-rated health. Human activity and behavior are the expressions and feedback of human mobility and experience in the spatial and temporal dimensions. The frequency of physical activity and travel mode variables used in this study are a quantitative spatial and temporal portrayal of the mobility of the human subject compared with the effects of objective physical space such as human testing indicators and the living environment on health from a ‘behavioral perspective’. This notion complements the ‘social-economic-environmental’ framework of health impacts. Exploring the coupling between human behavior and health is also challenging. Although the continuous development of GIS and spatial positioning technology has made it possible to monitor microscopic human mobility trajectories and their spatial and temporal characteristics, and to improve the accuracy of behavioral trajectory monitoring, it is still based on small samples and small areas. Differences in the privacy and security requirements of different groups of people can also result in limitations in the types of samples to be monitored and tracked. Reflective scales are mainly an individual’s overall evaluation of behavioral activities and the health assessment, real-time feedback, or recall assessment of objective facts, with the advantage of simplicity and speed, wide use, and a true reflection of objective concepts to some extent. Accordingly, the reflective scale questionnaire in this study still has the advantage of being used in a large sample of large regions on a national scale and further validates the accuracy and scientific validity of the result that ‘activity preference and travel behavior’ have a significant effect on health. The negative health effect of the travel mode variable further suggests that motorized travel helps to avoid the risk of exposure to ambient environmental pollution, thus improving subjective perceived health.

### 5.4. An Important Strategy to Improve the Health of the Population

#### 5.4.1. Positive Realization of the Healthy City Plan

In the rapid urbanization process, urban planning to further strengthen environmental protection and environmental pollution control and improve the quality of the residents’ living environment is significantly important to improve the health of the residents. At present, environmental pollution such as air and water pollution, caused by urban expansion, is a serious threat to the physical and psychological health of residents. Many Chinese cities have gradually emphasized that “along with China’s rapid industrialization, pollution of the air, water, soil, and other ecological environments, as well as food and drug safety issues, constitute a major health hazard for the nation”. The Healthy China strategy is an important element of China’s basic national development strategy, and healthy cities are a major goal that Chinese cities are striving to achieve. In 2013, 2018, and 2021, cities have continued to emphasize the major strategy of “fighting the battle against pollution”, promoting urban planning to strengthen “environmental protection and governance”. At present, urban planning has achieved good results in the planning, management, and remediation of environmental pollution issues such as air and water pollution. Food safety is one of the most important urban issues for urban management and is a key point for the health of residents. To further improve the health of the population, it is therefore of great theoretical and practical importance to study and implement strategies for healthy city planning that address a range of issues such as air pollution, water pollution, and food safety.

#### 5.4.2. Enhancing the Scale and Accessibility of Blue-Green Spaces

Enhancing the scale of urban green space improves the accessibility of green space for residents, so urban and rural residents can have access to sufficient green space. The urban blue-green space can provide residents with places to exercise, which is conducive to improving their physical health; at the same time, the blue-green space also has a significant psychological health-enhancing effect. The urban blue-green space has a significant PM2.5 reduction effect. Green spaces mitigate PM2.5 pollution through their open space characteristics and biological purification functions, whereas blue spaces reduce PM2.5 concentrations due to their good ventilation and diffusion functions. In urban landscape planning and green space system planning, the scale of green space and green space coverage can be further enhanced. More blue and green spaces as dots and strips can be distributed in high-density urban spaces, which can improve their spatial utilization efficiency and health effects. The diversity of land use types can be improved, and a reasonable scale of blue-green space can be allocated in residential and working areas.

#### 5.4.3. Scientific Mechanisms for Physical Exercise

① Further increase the frequency of physical activity where conditions allow. Whereas spatial diversity and green space accessibility can enhance a healthy quality of life, this depends on the individual’s walkability choices and frequency of walking. Therefore, increasing the frequency of physical activity can further improve people’s health, satisfaction, and well-being. ② The choice of open spaces or parks as places of activity is a good indicator of the pollution-reducing effect of blue-green urban spaces, which allow residents to breathe fresh air when exercising. This choice avoids areas with high air pollution such as near city roads and along the road during peak commuting hours. The area near city roads is a notable area for the accumulation of vehicle emissions, which makes it easier to inhale harmful substances when doing physical exercise, which is not good for one’s health. ③ Make scientific travel and exercise plans, use environmental pollution monitoring data, focus on the level of air pollution, water pollution, and other environmental problems in activity in advance, and avoid long-term exposure to areas with serious air pollution. ④ Monitor the physical exercise process for any discomfort, shortness of breath, coughing, and other problems.

#### 5.4.4. Bridging the Gap between Urban and Rural Areas and Increasing Residents’ Incomes

① Actively promote urban renewal, improve urban governance, renovate dilapidated and decaying facilities in the city, and build a safer and more comfortable public space environment. This will help improve the health of residents by attracting more participation in public activities. ② Retrofit communities with age-appropriate facilities, select safe and suitable locations for elderly people’s activities and encourage them to participate in physical exercise. ③ Based on the characteristic resources of the countryside, promote the development of rural industries, optimize the industrial layout, increase the income level of farmers, and improve their income level. Scientifically lay out the ecological space for production and living in the countryside, preserve as much of the original landforms and natural ecology as possible, systematically protect the natural scenery and idyllic landscapes of the countryside, and improve its appearance. ④ Improve and improve the living environment in economically disadvantaged areas, narrow the gap between urban and rural areas, and compensate for the shortcomings in infrastructure and public service facilities in economically disadvantaged areas. ⑤ Insist on the integration of urban and rural areas, reflecting the distinction between urban and rural areas, and design different development strategies for different types of villages according to local conditions. Focus on the common construction and sharing of infrastructure and public service facilities between villages, and strengthen the farmers’ service function of villages.

## 6. Conclusions

This study used big data from dynamic air quality monitoring and social survey data (e.g., the China CGSS National Survey) to assess the spatial differences and coupling relationships between subjective and objective air pollution. Furthermore, this study explored the mechanisms of social inequality, air pollution, food security, and insufficient green space for health, based on a logistic model. The study found that:

(1) Significant heterogeneity was observed between subjective and objective air pollution. The correlation coefficient between subjective and objective air pollution was small and the internal association between the two was insignificant. The significance of the subjective and objective air pollution variables in the logistic model was different, with the subjective variables being significant, whereas the objective variables were excluded from the model variables. Thus, subjective air pollution has a more significant influence on the residents’ health.
(2) Based on a health study of a complete sample of urban and rural residents, income inequality, air pollution, food pollution, and travel behavior can significantly affect the health level of residents, and the negative health effects of environmental pollution from air pollution, food pollution, and insufficient green space are evident. Furthermore, urban–rural health inequalities from the perspective of socioeconomic inequalities are also particularly evident, with gender, household size, travel, and physical activity having insignificant effects on the health of the rural population. Health improvement from increased income is much higher for groups with lower income levels than for those with higher income levels. The health-enhancing benefits per unit of income are much higher for rural residents than for urban residents.
(3) This study found a significant urban–rural differentiation mechanism for environmental health effects from a health perspective. Logistic regressions were conducted on urban and rural samples to determine the degree of influence and significance of the variables between them based on the coefficients. The results indicate that urban residents are more concerned about health from an environmental quality perspective and are more at risk from air pollution and insufficient green space elements. In this study, food pollution variables were included in the independent variable system of the regression equation to explore the health impact factors, and a negative effect of food pollution on health was found. Furthermore, rural residents were more likely to have negative health effects when affected by food pollution due to the urban–rural divide in healthy food desertification, and their risk of exposure to food pollution exposure was greater. In this study, food contamination variables were included in the independent variable system of the exploratory regression equation for factors that influence health, and negative health effects of food contamination were found. Furthermore, an urban–rural health inequality differentiation mechanism caused by the urban–rural divide of healthy food desertification was found.

In summary, ensuring social parity, strict control of environmental pollution, healthy exercise, and travel can help narrow the gap between rich and poor, promote urban–rural health equity, and improve human health in China.

## Figures and Tables

**Table 1:** Variable assignment.

Variable	Symbol 1	Symbol 2	Description	Relevance
Health level	Health	a16a		1
Annual income	Income	a8a	Individual total annual income of last year (2012)	+
Urban–rural	Urban	s5a	Urban = 1 and rural = 0	+
Region	Province	s41	Survey area (province)	
Age	Age	a3aa	Age	−
Marriage	Marriage	a69	Unmarried = 1; cohabiting = 2; first married with a spouse = 3; remarried with a spouse = 4; separated and not divorced = 5; divorced = 6; widowed = 7	−
Stratum	Stratum	a43a	The highest ‘10 points’ represents the top stratum, and the lowest ‘1 point’ represents the bottom stratum.	+
Education	Education	a7a	1 = no education; 2 = private school and literacy classes; 3 = primary school; 4 = junior high school; 5 = vocational high school; 6 = general high school; 7 = secondary school; 8 = technical school; 9 = university specialist (adult higher education); 10 = university specialist (formal higher education); 11 = university undergraduate (adult higher education); 12 = university undergraduate (formal higher education); 13 = graduate; 14 = doctoral students and above.	+
Gender	Sex	a2	1 = male and 2 = female	−
Household size	Family size	a63	How many people usually live in your household at the moment (including yourself)	−
Housing area per capita	Housing area per capita	a11	Housing area per capita	+
Frequency of physical exercise	Frequency of physical exercise	a3009	1 = daily; 2 = several times a week; 3 = several times a month; 4 = several times a year or less; 5 = never	−
Travel mode	Travel mode	b1105	I always take a taxi or private car when I go out 1 = very much so; 2 = more so; 3 = not very much so; and 4 = very little so	−
Air pollution	Air pollution	b21b01	1 = very serious; 2 = more serious; 3 = less serious; 4 = not serious; 5 = average; and 6 = no such problem	+
Water pollution	Water pollution	b21b02	1 = very serious; 2 = more serious; 3 = less serious; 4 = not serious; 5 = average; and 6 = no such problem	+
Noise pollution	Noise pollution	b21b03	1 = very serious; 2 = more serious; 3 = less serious; 4 = not serious; 5 = average; and 6 = no such problem	+
Food pollution	Food contamination	b21b10	1 = very serious; 2 = more serious; 3 = less serious; 4 = not serious; 5 = average; and 6 = no such problem	+
Lack of green space	Insufficient green space	b21b06	1 = very serious; 2 = more serious; 3 = less serious; 4 = not serious; 5 = average; and 6 = no such problem	+
Degradation of arable land quality	Degradation of cultivated land quality	b21b08	1 = very serious; 2 = more serious; 3 = less serious; 4 = not serious; 5 = average; and 6 = no such problem	+

Note: “+” is positive correlation, and ”−“ is negative correlation.

**Table 2:** Statistical description of variables.

Variable	Symbol	Maximum	Minimum	Average
Health level	a16a	1	0	0.8
Annual income	a8a	1,000,000	0	28,365.04
Urban–rural	s5a	5	1	2.691315
Age	a3aa	96	17	46.45288
Marriage	a69	7	1	3.092723
Stratum	a43a	10	1	4.459759
Education	a7a	14	1	5.661469
Gender	a2	2	1	1.456908
Household size	a63	12	1	3.094903
Housing area per capita	a11a	700	1.5	41.57517
Frequency of physical exercise	a3009	5	1	3.719651
Travel mode	b1105	4	1	3.307344
Air pollution	b21b01	6	1	3.348759
Water pollution	b21b02	6	1	3.473587
Food contamination (food safety)	b21b10	6	1	3.682428
Lack of green space	b21b06	6	1	4.129297

**Table 3:** Comparison of the full-sample multi-model regression results.

Variable	Code	Model 1		Model 2		Model 3	
		Coe. b	Sig.	Coe. b	Sig.	Coe. b	Sig.
Gender	a2	−0.153	0.034 **	−0.154	0.033 **	−0.056	0.000 ***
Age	a3aa	−0.046	0 ***	−0.046	0.000 ***	−0.017	0.019 **
Urban-rural	s5aa	0.189	0.034 **	0.204	0.020 **	0.096	0.000 ***
Stratum	a43a	0.16	0 ***	0.159	0.000 ***	0.058	0.001 ***
Household size	a63	0.046	0.098 *	0.045	0.109	0.020	0.000 ***
Education	a7a	0.06	0 ***	0.060	0.000 ***	0.017	0.036 **
Income	a8aaa	0.294	0.08 *	0.296	0.078 *	0.080	0.000 ***
Area per capita	a11aa	0.051	0.016 **	0.049	0.019 **	0.015	0.011 **
Frequency of physical exercise	a3009	−0.093	0 ***	−0.094	0.000 ***	−0.062	0.034 **
Travel mode	b1105	−0.125	0.013 **	−0.130	0.009 ***	−0.039	0.000 ***
Lack of green space	b21b06	0.052	0.012 **	0.045	0.024 **	0.015	0.009 ***
Food contamination (food safety)	b21b08	0.02	0.173 **	0.018	0.224	0.003	0.013 **
East and West	s41 East and West	0.318	0 ***	0.264	0.006 ***	0.040	0.384
Air pollution (subjective)	S6kq	0.024	0.025 **	-	-	0.000	0.165
PM2.5 (objective)	PM2.5	-		0.003	0.216	−0.002	0.978
Constants	e	2.577	0 ***	2.678	0.000 ***	4.729	0.000 ***
ROC AUC		0.854		0.806		0.837	
Predicted correct rate		88.261		80.345		85.543	
AIC		5270		4988		-	

Note: ***, **, and * indicate significance at the 1%, 5%, and 10% levels, respectively. Model 1 is the logistic model with subjective air pollution. Model 2 is the logistic model with the objective of air pollution. Model 3 is a linear model. Abbreviations: PM2.5, particulate matter 2.5 μm or less; ROC AUC, Receiver operating characteristic area under curve; AIC, Akaike information criterion.

**Table 4:** A comparison of the regression results for urban–rural differences in the whole sample.

Variables	Code	Model 1		Model 4		Model 5	
		Coe. b	Sig.	Coe. b	Sig.	Coe. b	Sig.
Gender	a2	−0.153	0.034 **	−0.196	0.031 **	0.041	0.740
Age	a3aa	−0.046	0.000 ***	−0.045	0.000 ***	−0.042	0.000 ***
Urban–rural	s5aa	0.189	0.034 **	— —	— —	— —	— —
Stratum	a43a	0.16	0 ***	0.178	0.000 ***	0.120	0.001 ***
Household size	a63	0.046	0.098 *	0.042	0.259	0.044	0.300
Education	a7a	0.06	0 ***	0.050	0.005 ***	0.114	0.003 ***
Income	a8aaa	0.294	0.08 *	0.053	0.074 *	2.161	0.000 ***
Area per capita	a11aa	0.051	0.016 **	0.029	0.341	0.064	0.032 **
Frequency of physical exercise	a3009	−0.093	0 ***	−0.103	0.001 ***	−0.051	0.358
Travel mode	b1105	−0.125	0.013 **	−0.165	0.006 ***	−0.016	0.858
Lack of green space	b21b06	0.052	0.012 **	0.058	0.052 *	0.051	0.101
Food contamination (food safety)	b21b08	0.02	0.173 **	0.017	0.036 **	0.027	0.330
East and West	s41 East and West	0.318	0 ***	0.326	0.004 ***	0.266	0.027 **
Air pollution	S6kq	0.024	0.025 **	0.026	0.032 **	0.025	0.141
Constants	e	2.577	0 ***	3.072	0.000 ***	1.251	0.071 *
ROC AUC		0.854		0.813		0.727	
Predicted correct rate		88.261		82.534		75.457	
AIC		5270		3337		1942	

Note: ***, **, and * indicate significance at the 1%, 5%, and 10% levels, respectively. Model 1 is the full-sample. Model 4 is the urban sample. Model 5 is the rural sample. Abbreviations: PM2.5, particulate matter of 2.5 μm or less; ROC AUC, receiver operating characteristic area under curve; AIC, Akaike information criterion.

## Data Availability

Restrictions apply to the availability of data. Data were obtained from the China Comprehensive Social Survey (CGSS).
